# Supplementary material for: Exploiting Oriented Field Projectors to Open Topological Gaps in Plasmonic Nanoparticle Arrays
Source: ACS Photonics. 2023 Jan 11;10(2):464–74. doi: 10.1021/acsphotonics.2c01526 (PMC10664046; doi:10.1021/acsphotonics.2c01526)
Supplement: Supplementary file 1 — ph2c01526_si_002.pdf [file ph2c01526_si_002.pdf]

SUPPORTING INFORMATION

Exploiting oriented field projectors to open topological gaps in plasmonic nanoparticle arrays

Álvaro Buendía, Jose A. Sánchez-Gil and Vincenzo Giannini

Comprised of
4 pages
0 figures
0 tables

Supporting Information: Exploiting oriented field projectors to open topological gaps in plasmonic nanoparticle arrays

Álvaro Buendía,[*,†] Jose A. Sánchez-Gil,[†] and Vincenzo Giannini[*,†,‡,¶]

†*Instituto de Estructura de la Materia, Consejo Superior de Investigaciones Científicas, Serrano 121, 28006 Madrid, Spain*

‡*Centre of Excellence ENSEMBLE3 sp. z o.o., Wolczynska 133, Warsaw, 01-919, Poland*

¶*Technology Innovation Institute, Masdar City 9639, Abu Dhabi, United Arab Emirates*

E-mail: a.buendia@csic.es; v.giannini@csic.es

Polarizability of metallic nanospheres and nanospheroids

Generally, the electric polarizability $\overleftrightarrow{\alpha}(\omega)$ acts like a tensor:

$$\overleftrightarrow{\alpha}(\omega) = \begin{pmatrix} \alpha_{xx} & \alpha_{xy} & \alpha_{xz} \\ \alpha_{xy} & \alpha_{yy} & \alpha_{yz} \\ \alpha_{xz} & \alpha_{yz} & \alpha_{zz} \end{pmatrix}. \tag{1}$$

For a nanosphere, due to the spherical symmetry, the polarizability is proportional to the identity matrix, $\overleftrightarrow{\alpha}(\omega) = \alpha(\omega)I$. When we are working in the limit $a \gg \lambda$ we can take the quasi-static approximation, assuming only the first Mie coefficient contributes to the

polarizability, so $\alpha(\omega)$ is:

$$\alpha(\omega) = 4\pi a^3 \epsilon_0 \frac{\epsilon(\omega) - \epsilon_\mathrm{B}}{\epsilon(\omega) + 2\epsilon_\mathrm{B}}. \tag{2}$$

The permittivity of the medium, $\epsilon(\omega)$ can be approximated by a Drude-Lorentz model:[1]

$$\epsilon(\omega) = \epsilon_r - \sum_j \frac{\omega_{\mathrm{P},j}^2}{\omega(\omega + i\gamma_j)} - \sum_j \frac{\Delta\epsilon_j \Omega_j^2}{\omega^2 - \Omega_j^2 + i\omega\Gamma_j}, \tag{3}$$

where ϵ_r is the static dielectric constant, $\omega_{\mathrm{P},j}$ are plasma frequencies, γ_j and Γ_j are the damping constants, Ω_j are resonant frequencies, and $\Delta\epsilon_j$ are related to the oscillator strengths. In our work we use the parameters for silver: $\epsilon_r = 4.6,\ \omega_{\mathrm{P},0} = 9.0,\ \gamma_0 = 0.07,\ \Gamma_0 = 1.2,\ \Omega_0 = 4.9,\ \Delta\epsilon_0 = 1.10.$[1]

The extinction cross section is the sum of absorption and scattering cross sections and for a single nanoparticle as given by:[2]

$$\sigma_\mathrm{ext} = \sigma_\mathrm{abs} + \sigma_\mathrm{sca} = \frac{k}{\epsilon_0}\mathrm{Im}(\alpha(\omega)) + \frac{k^4}{6\pi\epsilon_0^2}|\alpha(\omega)|^2. \tag{4}$$

Now we consider prolate spheroids, that is, ellipsoids with major axis a and minor axes $b = c$. The polarizability doesn't behave like a scalar anymore, but depends on the polarization of the incoming electric field. The polarizabilities for the main axes, $\alpha_l(\omega)$ with $l \in [a, b, c]$, are:[3]

$$\alpha_l(\omega) = V \frac{\epsilon(\omega) - \epsilon_b}{\epsilon_b + L_l(\epsilon(\omega) - \epsilon_b)}, \tag{5}$$

V being the volume of the spheroid, $V = \frac{4}{3}\pi a c^2$, and L_l are geometric factors given by:

$$L_a = \frac{e^2}{1 - e^2}\left(\frac{1}{2\sqrt{1-e^2}} \ln\left(\frac{1+\sqrt{1-e^2}}{1-\sqrt{1-e^2}}\right) - 1\right),$$
$$L_b = L_c = \frac{1 - L_a}{2}, \tag{6}$$

where $e = c/a$ is the eccentricity of the spheroid, ranging from $e = 0$ (needle) to $e = 1$

(sphere).

Let us now consider an array of nanoparticles, where the major axis of the spheroid n is in the direction $\mathbf{u}_n = (\sin\theta_n \cos\varphi_n, \sin\theta_n \sin\varphi_n, \cos\theta_n)$, where θ_n and φ_n are the angles formed by the spheroidal major axis with respect to the z and x axis. When $q \ll 1$, in the vicinity of the major axis resonance ω_{spa}, $\alpha_c(\omega \simeq \omega_{spa}) \simeq 0$, we can approximate the polarizability tensor as:

$$\overleftrightarrow{\alpha_n}(\omega) \simeq \alpha_a(\omega) \begin{pmatrix} \sin^2\theta_n \cos^2\varphi_n & \sin^2\theta_n \sin\varphi_n \cos\varphi_n & \sin\theta_n \cos\theta_n \cos\varphi_n \\ \sin^2\theta_n \sin\varphi_n \cos\varphi_n & \sin^2\theta_n \sin^2\varphi_n & \sin\theta_n \cos\theta_n \sin\varphi_n \\ \sin\theta_n \cos\theta_n \cos\varphi_n & \sin\theta_n \cos\theta_n \sin\varphi_n & \cos^2\theta_n \end{pmatrix}, \quad (7)$$

which projects any vector $\mathbf{v}$ in the direction $\mathbf{u}_n$, i.e. $\overleftrightarrow{\alpha}(\omega)\mathbf{v} = \alpha_a(\omega)(\mathbf{v} \cdot \mathbf{u}_n) \cdot \mathbf{u}_n$.

As the direction of the dipoles is fixed, we can project $\mathbf{p}_n$ in the direction u_n, so we get the scalar coupled-dipole equations in main text.

Green dyadic's function projection

As we saw in last section, near ω_{spa} the polarizabilities of the nanospheroids project the dipoles in the directions of the major axes. This turns in scalar coupled-dipole equations. Explicitly, the Green's dyadic function projection for two nanospheroids with major axes oriented in $\mathbf{u}_n = (\sin\theta_n \cos\varphi_n, \sin\theta_n \sin\varphi_n, \cos\theta_n)$ and $\mathbf{u}_m = (\sin\theta_m \cos\varphi_m, \sin\theta_m \sin\varphi_m, \cos\theta_m)$ directions, $G_{\mathbf{u}_m,\mathbf{u}_n}$, is:

$$\begin{aligned} G_{\mathbf{u}_m,\mathbf{u}_n} = (\overleftrightarrow{\mathbf{G}}(\mathbf{r}_m, \mathbf{r}_n, \omega) \cdot \mathbf{u}_m) \cdot \mathbf{u}_n = \sum_{\mu,\nu} G_{\mu\nu} [\mathbf{u}_n]_\mu [\mathbf{u}_n]_\nu = \\ = G_{xx} \cos\varphi_m \cos\varphi_n \sin\theta_m \sin\theta_n + G_{xy} \sin\theta_m \sin\theta_n \sin(\varphi_m + \varphi_n) + \\ G_{xz} (\cos\theta_m \cos\varphi_n \sin\theta_n + cos\theta_n \cos\varphi_m \sin\theta_m) + G_{yy} \sin\varphi_m \sin\varphi_n \sin\theta_m \sin\theta_n + \\ + G_{yz} (\cos\theta_m \sin\varphi_n \sin\theta_n + cos\theta_n \sin\varphi_m \sin\theta_m) + G_{zz} \cos\theta_m \cos\theta_n, \end{aligned} \quad (8)$$

where $\mu, \nu = x, y, z$ are the polarizations and $G_{\mu\nu}$ are the elements of the Green dyadic $\overleftrightarrow{\mathbf{G}}(\mathbf{r}_m, \mathbf{r}_n, \omega)$.

For a linear array of nanoparticles along the x direction, EQ. 8 reduces to:

$$G_{\mathbf{u}_m,\mathbf{u}_n} = \frac{(2\cos\varphi_m\cos\varphi_n - \sin\varphi_m\sin\varphi_n)\sin\theta_m\sin\theta_n - \cos\theta_m\cos\theta_n}{4\pi k^2 R^3}, \tag{9}$$

which for $\theta_n = \theta_m = \frac{\pi}{2}$ derives in the equation for the projection of the Green's function in main text. In this paper, we restricted the orientations of the dipoles to the xy or xz planes, but by orienting them in the space, we could add another extra degree of freedom.

References

(1) Rodrigo, S. G.; García-Vidal, F. J.; Martín-Moreno, L. Influence of material properties on extraordinary optical transmission through hole arrays. *Phys. Rev. B* **2008**, *77*, 075401.

(2) Novotny, L.; Hecht, B. *Principles of Nano-Optics*, 2nd ed.; Cambridge University Press, 2012.

(3) Moroz, A. Depolarization field of spheroidal particles. *Journal of the Optical Society of America B* **2009**, *26*, 517.
